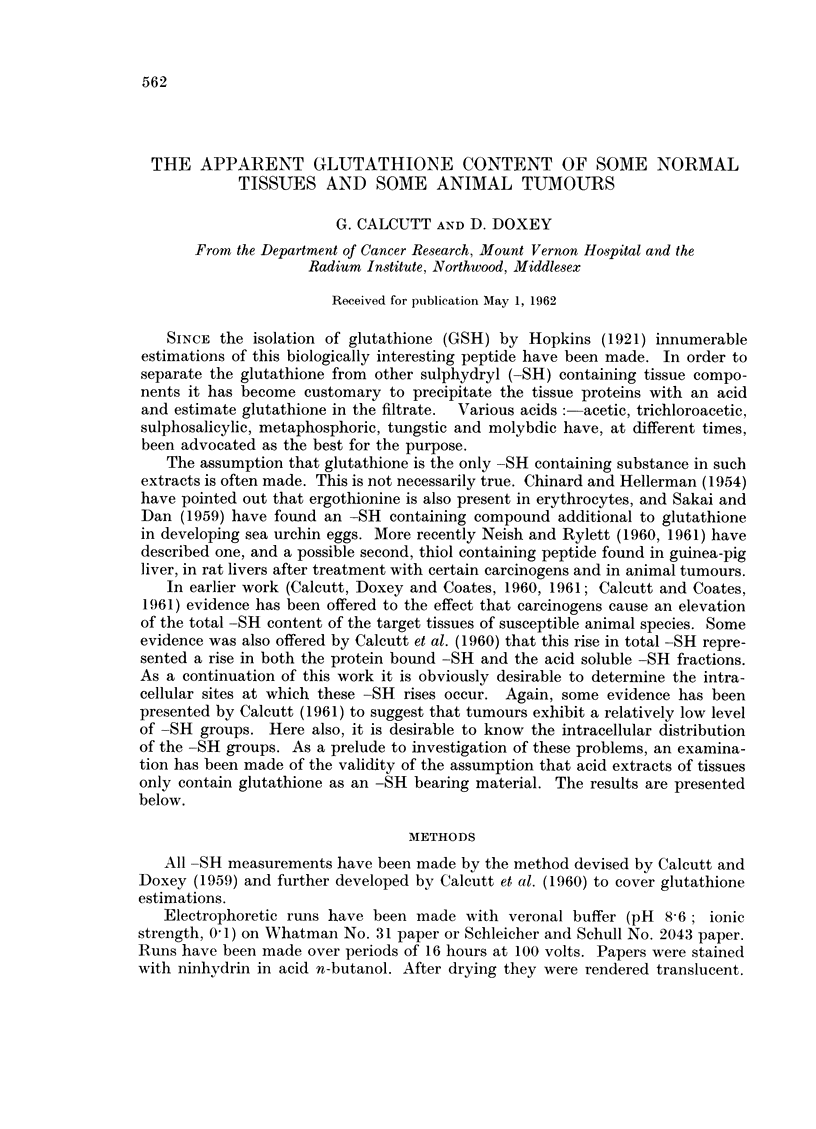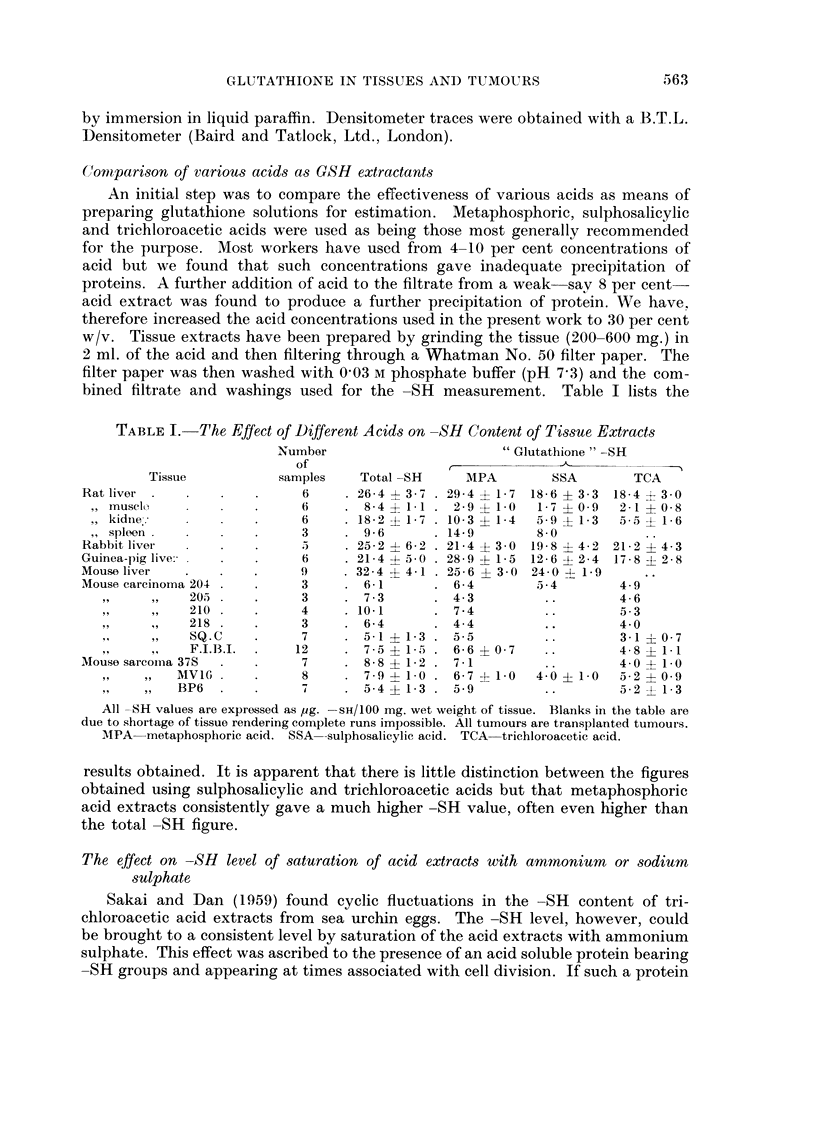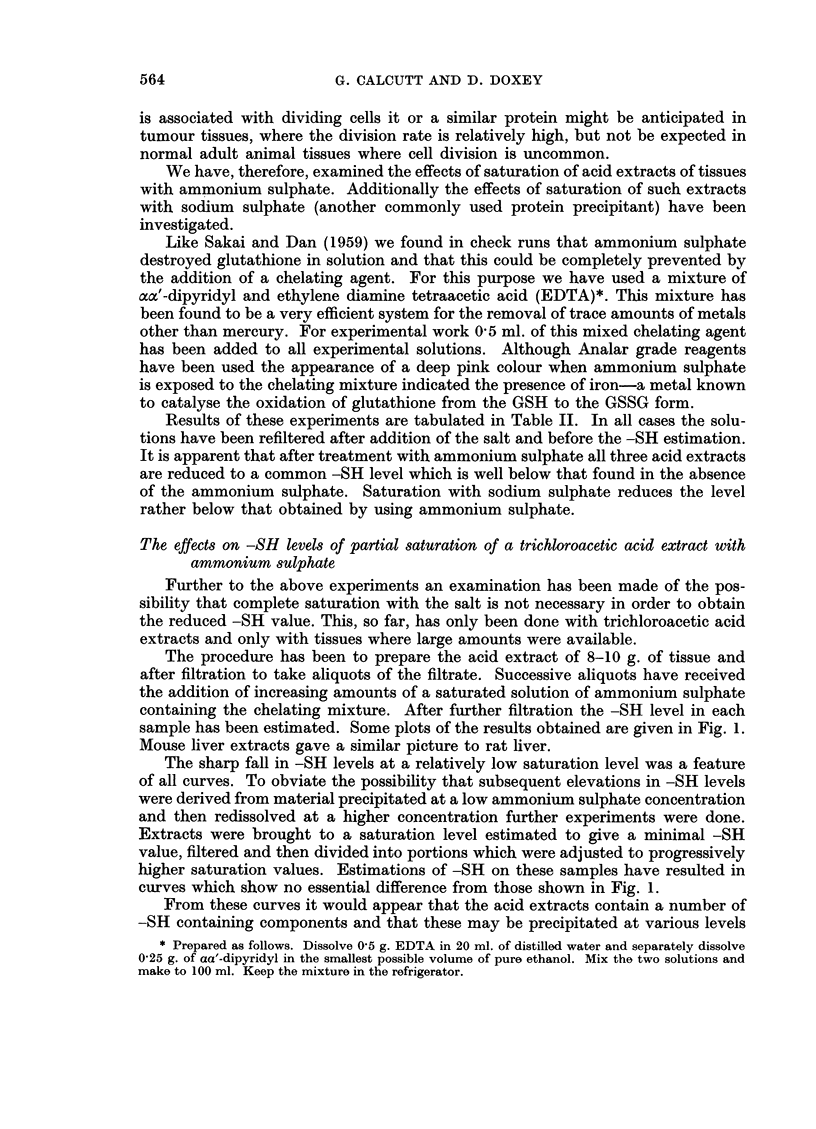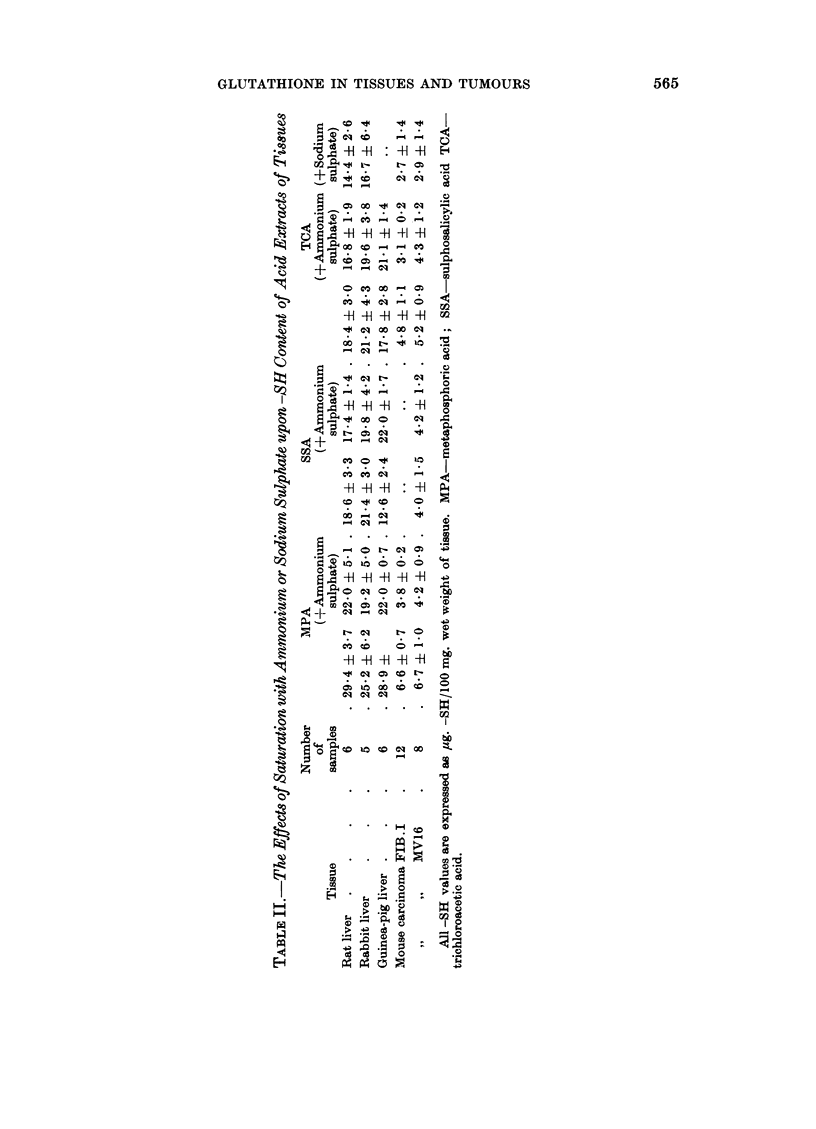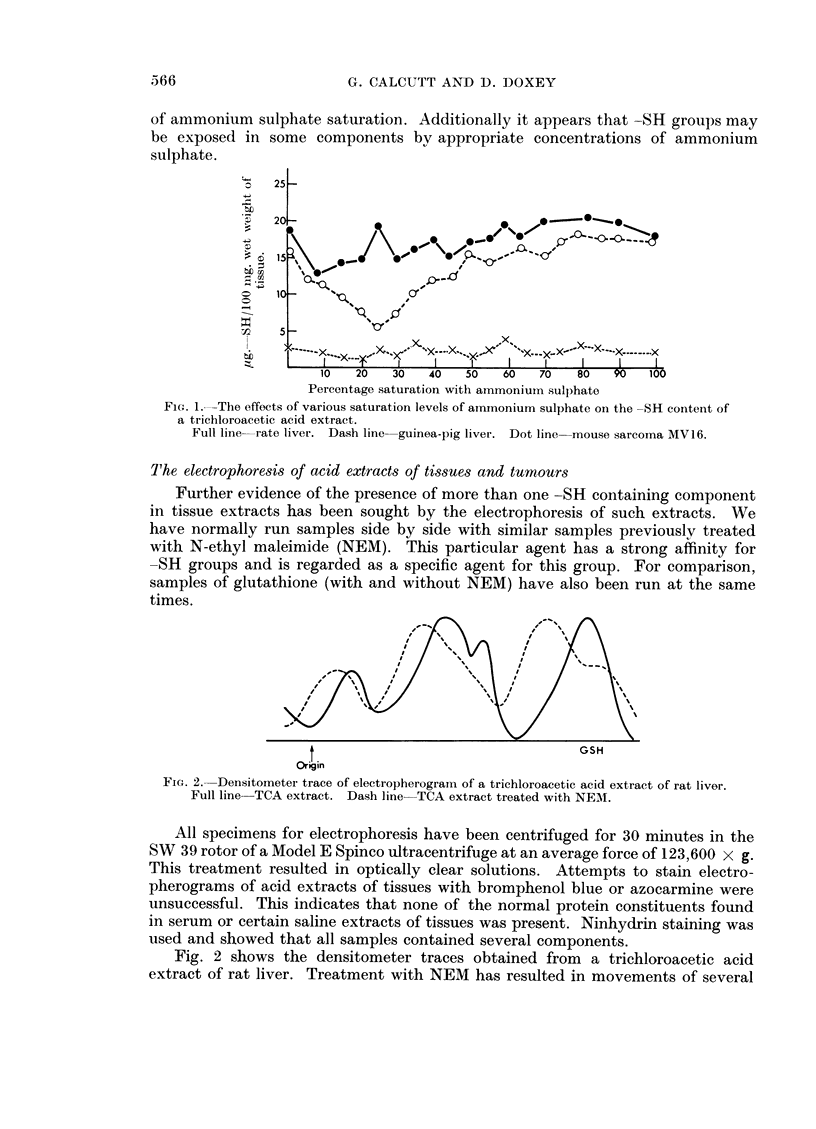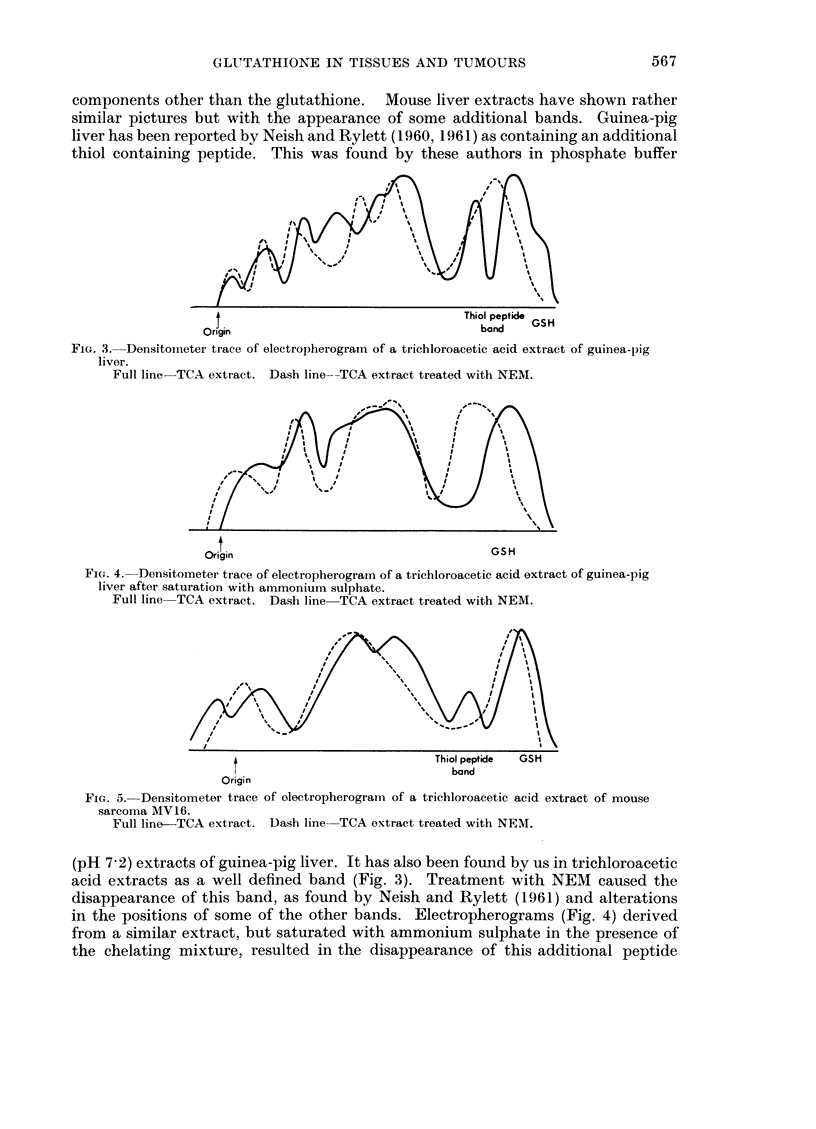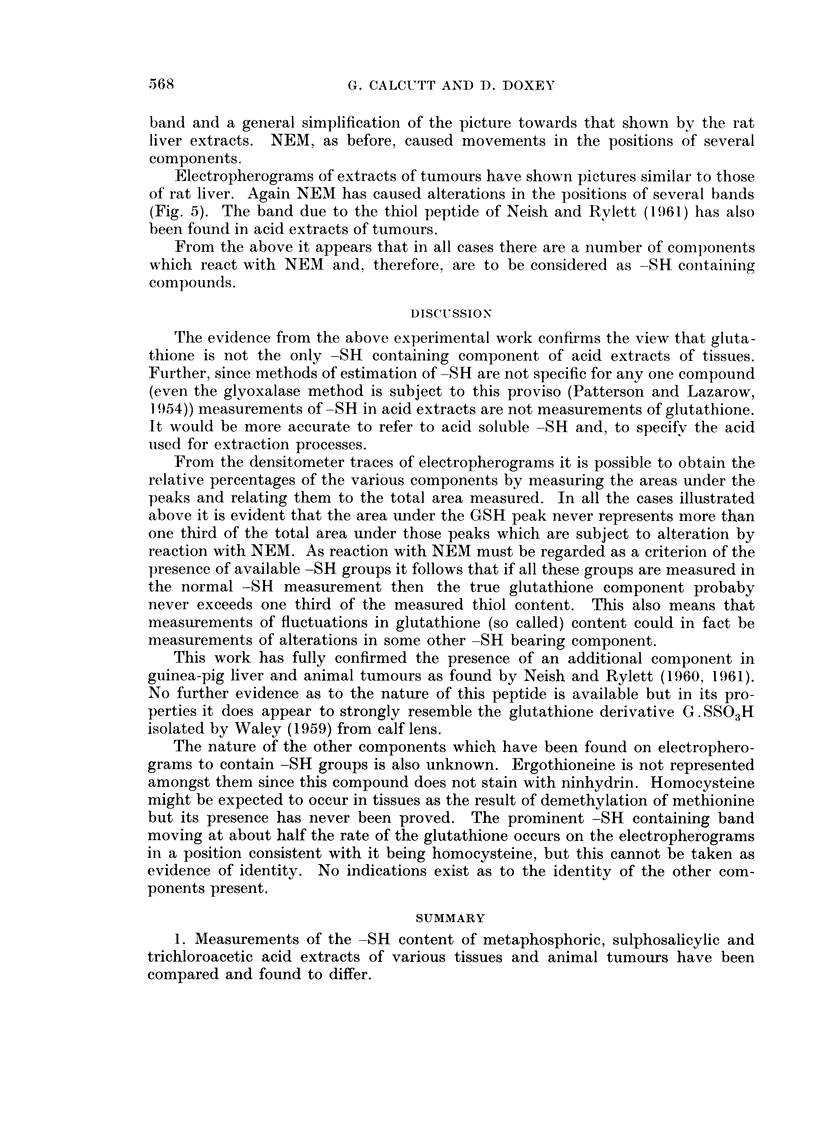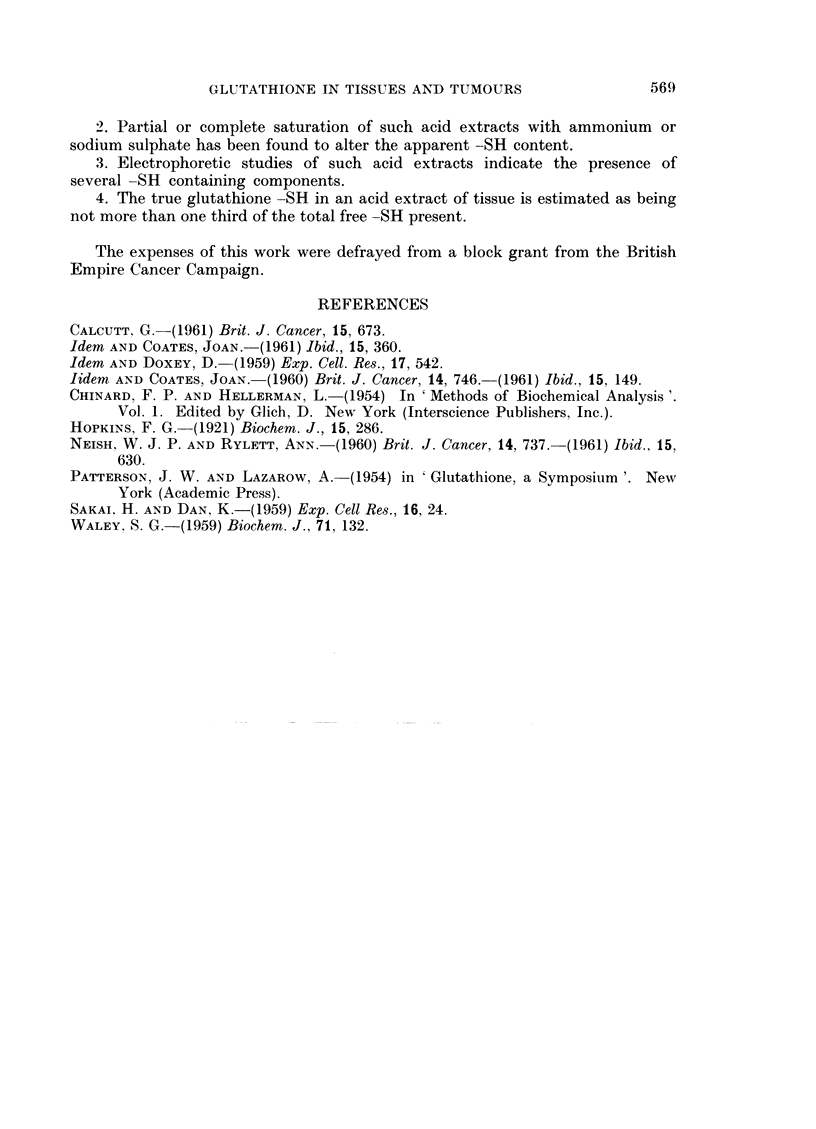# The Apparent Glutathione Content of Some Normal Tissues and some Animal Tumours

**DOI:** 10.1038/bjc.1962.65

**Published:** 1962-09

**Authors:** G. Calcutt, D. Doxey


### THE APPARENT GLUTATHIONE CONTENT OF SOME NORMAL TISSUES AND SOME ANIMAL TUMOURS

G. CALCUTT AND D. DOXEY

*From the Department of Cancer Research, Mount Vernon Hospital and the Radium Institute, Northwood, Middlesex*

Received for publication May 1, 1962

SINCE the isolation of glutathione (GSH) by Hopkins (1921) innumerable estimations of this biologically interesting peptide have been made. In order to separate the glutathione from other sulphydryl (–SH) containing tissue components it has become customary to precipitate the tissue proteins with an acid and estimate glutathione in the filtrate. Various acids :—acetic, trichloroacetic, sulphosalicylic, metaphosphoric, tungstic and molybdic have, at different times, been advocated as the best for the purpose.

The assumption that glutathione is the only –SH containing substance in such extracts is often made. This is not necessarily true. Chinard and Hellerman (1954) have pointed out that ergothionine is also present in erythrocytes, and Sakai and Dan (1959) have found an –SH containing compound additional to glutathione in developing sea urchin eggs. More recently Neish and Rylett (1960, 1961) have described one, and a possible second, thiol containing peptide found in guinea-pig liver, in rat livers after treatment with certain carcinogens and in animal tumours.

In earlier work (Calcutt, Doxey and Coates, 1960, 1961; Calcutt and Coates, 1961) evidence has been offered to the effect that carcinogens cause an elevation of the total –SH content of the target tissues of susceptible animal species. Some evidence was also offered by Calcutt *et al.* (1960) that this rise in total –SH represented a rise in both the protein bound –SH and the acid soluble –SH fractions. As a continuation of this work it is obviously desirable to determine the intracellular sites at which these –SH rises occur. Again, some evidence has been presented by Calcutt (1961) to suggest that tumours exhibit a relatively low level of –SH groups. Here also, it is desirable to know the intracellular distribution of the –SH groups. As a prelude to investigation of these problems, an examination has been made of the validity of the assumption that acid extracts of tissues only contain glutathione as an –SH bearing material. The results are presented below.

### METHODS

All –SH measurements have been made by the method devised by Calcutt and Doxey (1959) and further developed by Calcutt *et al.* (1960) to cover glutathione estimations.

Electrophoretic runs have been made with veronal buffer (pH 8·6 ; ionic strength, 0·1) on Whatman No. 31 paper or Schleicher and Schull No. 2043 paper. Runs have been made over periods of 16 hours at 100 volts. Papers were stained with ninhydrin in acid *n*-butanol. After drying they were rendered translucent.

by immersion in liquid paraffin. Densitometer traces were obtained with a B.T.L. Densitometer (Baird and Tatlock, Ltd., London).

*Comparison of various acids as GSH extractants*

An initial step was to compare the effectiveness of various acids as means of preparing glutathione solutions for estimation. Metaphosphoric, sulphosalicylic and trichloroacetic acids were used as being those most generally recommended for the purpose. Most workers have used from 4–10 per cent concentrations of acid but we found that such concentrations gave inadequate precipitation of proteins. A further addition of acid to the filtrate from a weak—say 8 per cent—acid extract was found to produce a further precipitation of protein. We have, therefore increased the acid concentrations used in the present work to 30 per cent w/v. Tissue extracts have been prepared by grinding the tissue (200–600 mg.) in 2 ml. of the acid and then filtering through a Whatman No. 50 filter paper. The filter paper was then washed with 0·03 M phosphate buffer (pH 7·3) and the combined filtrate and washings used for the –SH measurement. Table I lists the results obtained. It is apparent that there is little distinction between the figures obtained using sulphosalicylic and trichloroacetic acids but that metaphosphoric acid extracts consistently gave a much higher –SH value, often even higher than the total –SH figure.

TABLE I.—*The Effect of Different Acids on –SH Content of Tissue Extracts*

| Tissue | Number of samples | Total –SH | "Glutathione" –SH MPA | SSA | TCA |
|---|---|---|---|---|---|
| Rat liver | 6 | $26·4 \pm 3·7$ | $29·4 \pm 1·7$ | $18·6 \pm 3·3$ | $18·4 \pm 3·0$ |
| ,, muscle | 6 | $8·4 \pm 1·1$ | $2·9 \pm 1·0$ | $1·7 \pm 0·9$ | $2·1 \pm 0·8$ |
| ,, kidney | 6 | $18·2 \pm 1·7$ | $10·3 \pm 1·4$ | $5·9 \pm 1·3$ | $5·5 \pm 1·6$ |
| ,, spleen | 3 | 9·6 | 14·9 | 8·0 | .. |
| Rabbit liver | 5 | $25·2 \pm 6·2$ | $21·4 \pm 3·0$ | $19·8 \pm 4·2$ | $21·2 \pm 4·3$ |
| Guinea-pig liver | 6 | $21·4 \pm 5·0$ | $28·9 \pm 1·5$ | $12·6 \pm 2·4$ | $17·8 \pm 2·8$ |
| Mouse liver | 9 | $32·4 \pm 4·1$ | $25·6 \pm 3·0$ | $24·0 \pm 1·9$ | .. |
| Mouse carcinoma 204 | 3 | 6·1 | 6·4 | 5·4 | 4·9 |
| ,, ,, 205 | 3 | 7·3 | 4·3 | .. | 4·6 |
| ,, ,, 210 | 4 | 10·1 | 7·4 | .. | 5·3 |
| ,, ,, 218 | 3 | 6·4 | 4·4 | .. | 4·0 |
| ,, ,, SQ.C | 7 | $5·1 \pm 1·3$ | 5·5 | .. | $3·1 \pm 0·7$ |
| ,, ,, F.I.B.I. | 12 | $7·5 \pm 1·5$ | $6·6 \pm 0·7$ | .. | $4·8 \pm 1·1$ |
| Mouse sarcoma 37S | 7 | $8·8 \pm 1·2$ | 7·1 | .. | $4·0 \pm 1·0$ |
| ,, ,, MV16 | 8 | $7·9 \pm 1·0$ | $6·7 \pm 1·0$ | $4·0 \pm 1·0$ | $5·2 \pm 0·9$ |
| ,, ,, BP6 | 7 | $5·4 \pm 1·3$ | 5·9 | .. | $5·2 \pm 1·3$ |

All –SH values are expressed as μg. —SH/100 mg. wet weight of tissue. Blanks in the table are due to shortage of tissue rendering complete runs impossible. All tumours are transplanted tumours.
MPA—metaphosphoric acid. SSA—sulphosalicylic acid. TCA—trichloroacetic acid.

*The effect on –SH level of saturation of acid extracts with ammonium or sodium sulphate*

Sakai and Dan (1959) found cyclic fluctuations in the –SH content of trichloroacetic acid extracts from sea urchin eggs. The –SH level, however, could be brought to a consistent level by saturation of the acid extracts with ammonium sulphate. This effect was ascribed to the presence of an acid soluble protein bearing –SH groups and appearing at times associated with cell division. If such a protein is associated with dividing cells it or a similar protein might be anticipated in tumour tissues, where the division rate is relatively high, but not be expected in normal adult animal tissues where cell division is uncommon.

We have, therefore, examined the effects of saturation of acid extracts of tissues with ammonium sulphate. Additionally the effects of saturation of such extracts with sodium sulphate (another commonly used protein precipitant) have been investigated.

Like Sakai and Dan (1959) we found in check runs that ammonium sulphate destroyed glutathione in solution and that this could be completely prevented by the addition of a chelating agent. For this purpose we have used a mixture of $\alpha\alpha'$-dipyridyl and ethylene diamine tetraacetic acid (EDTA)*. This mixture has been found to be a very efficient system for the removal of trace amounts of metals other than mercury. For experimental work 0·5 ml. of this mixed chelating agent has been added to all experimental solutions. Although Analar grade reagents have been used the appearance of a deep pink colour when ammonium sulphate is exposed to the chelating mixture indicated the presence of iron—a metal known to catalyse the oxidation of glutathione from the GSH to the GSSG form.

Results of these experiments are tabulated in Table II. In all cases the solutions have been refiltered after addition of the salt and before the –SH estimation. It is apparent that after treatment with ammonium sulphate all three acid extracts are reduced to a common –SH level which is well below that found in the absence of the ammonium sulphate. Saturation with sodium sulphate reduces the level rather below that obtained by using ammonium sulphate.

*The effects on –SH levels of partial saturation of a trichloroacetic acid extract with ammonium sulphate*

Further to the above experiments an examination has been made of the possibility that complete saturation with the salt is not necessary in order to obtain the reduced –SH value. This, so far, has only been done with trichloroacetic acid extracts and only with tissues where large amounts were available.

The procedure has been to prepare the acid extract of 8–10 g. of tissue and after filtration to take aliquots of the filtrate. Successive aliquots have received the addition of increasing amounts of a saturated solution of ammonium sulphate containing the chelating mixture. After further filtration the –SH level in each sample has been estimated. Some plots of the results obtained are given in Fig. 1. Mouse liver extracts gave a similar picture to rat liver.

The sharp fall in –SH levels at a relatively low saturation level was a feature of all curves. To obviate the possibility that subsequent elevations in –SH levels were derived from material precipitated at a low ammonium sulphate concentration and then redissolved at a higher concentration further experiments were done. Extracts were brought to a saturation level estimated to give a minimal –SH value, filtered and then divided into portions which were adjusted to progressively higher saturation values. Estimations of –SH on these samples have resulted in curves which show no essential difference from those shown in Fig. 1.

From these curves it would appear that the acid extracts contain a number of –SH containing components and that these may be precipitated at various levels

* Prepared as follows. Dissolve 0·5 g. EDTA in 20 ml. of distilled water and separately dissolve 0·25 g. of $\alpha\alpha'$-dipyridyl in the smallest possible volume of pure ethanol. Mix the two solutions and make to 100 ml. Keep the mixture in the refrigerator.

TABLE II.—*The Effects of Saturation with Ammonium or Sodium Sulphate upon –SH Content of Acid Extracts of Tissues*

| Tissue | Number of samples | MPA | MPA (+Ammonium sulphate) | SSA | SSA (+Ammonium sulphate) | TCA | TCA (+Ammonium sulphate) | TCA (+Sodium sulphate) |
|---|---|---|---|---|---|---|---|---|
| Rat liver | 6 | 29·4 ± 3·7 | 22·0 ± 5·1 | 18·6 ± 3·3 | 17·4 ± 1·4 | 18·4 ± 3·0 | 16·8 ± 1·9 | 14·4 ± 2·6 |
| Rabbit liver | 5 | 25·2 ± 6·2 | 19·2 ± 5·0 | 21·4 ± 3·0 | 19·8 ± 4·2 | 21·2 ± 4·3 | 19·6 ± 3·8 | 16·7 ± 6·4 |
| Guinea-pig liver | 6 | 28·9 ± | 22·0 ± 0·7 | 12·6 ± 2·4 | 22·0 ± 1·7 | 17·8 ± 2·8 | 21·1 ± 1·4 | .. |
| Mouse carcinoma FIB.I | 12 | 6·6 ± 0·7 | 3·8 ± 0·2 | .. | .. | 4·8 ± 1·1 | 3·1 ± 0·2 | 2·7 ± 1·4 |
| ,, ,, MV16 | 8 | 6·7 ± 1·0 | 4·2 ± 0·9 | 4·0 ± 1·5 | 4·2 ± 1·2 | 5·2 ± 0·9 | 4·3 ± 1·2 | 2·9 ± 1·4 |

All –SH values are expressed as μg. –SH/100 mg. wet weight of tissue. MPA—metaphosphoric acid; SSA—sulphosalicylic acid TCA—trichloroacetic acid.

of ammonium sulphate saturation. Additionally it appears that -SH groups may be exposed in some components by appropriate concentrations of ammonium sulphate.

Fig. 1.—The effects of various saturation levels of ammonium sulphate on the -SH content of a trichloroacetic acid extract.
Full line—rate liver. Dash line—guinea-pig liver. Dot line—mouse sarcoma MV16.

*The electrophoresis of acid extracts of tissues and tumours*

Further evidence of the presence of more than one -SH containing component in tissue extracts has been sought by the electrophoresis of such extracts. We have normally run samples side by side with similar samples previously treated with N-ethyl maleimide (NEM). This particular agent has a strong affinity for -SH groups and is regarded as a specific agent for this group. For comparison, samples of glutathione (with and without NEM) have also been run at the same times.

Fig. 2.—Densitometer trace of electropherogram of a trichloroacetic acid extract of rat liver.
Full line—TCA extract. Dash line—TCA extract treated with NEM.

All specimens for electrophoresis have been centrifuged for 30 minutes in the SW 39 rotor of a Model E Spinco ultracentrifuge at an average force of 123,600 × g. This treatment resulted in optically clear solutions. Attempts to stain electropherograms of acid extracts of tissues with bromphenol blue or azocarmine were unsuccessful. This indicates that none of the normal protein constituents found in serum or certain saline extracts of tissues was present. Ninhydrin staining was used and showed that all samples contained several components.

Fig. 2 shows the densitometer traces obtained from a trichloroacetic acid extract of rat liver. Treatment with NEM has resulted in movements of several components other than the glutathione. Mouse liver extracts have shown rather similar pictures but with the appearance of some additional bands. Guinea-pig liver has been reported by Neish and Rylett (1960, 1961) as containing an additional thiol containing peptide. This was found by these authors in phosphate buffer

FIG. 3.—Densitometer trace of electropherogram of a trichloroacetic acid extract of guinea-pig liver.
Full line—TCA extract. Dash line—TCA extract treated with NEM.

FIG. 4.—Densitometer trace of electropherogram of a trichloroacetic acid extract of guinea-pig liver after saturation with ammonium sulphate.
Full line—TCA extract. Dash line—TCA extract treated with NEM.

FIG. 5.—Densitometer trace of electropherogram of a trichloroacetic acid extract of mouse sarcoma MV16.
Full line—TCA extract. Dash line—TCA extract treated with NEM.

(pH 7·2) extracts of guinea-pig liver. It has also been found by us in trichloroacetic acid extracts as a well defined band (Fig. 3). Treatment with NEM caused the disappearance of this band, as found by Neish and Rylett (1961) and alterations in the positions of some of the other bands. Electropherograms (Fig. 4) derived from a similar extract, but saturated with ammonium sulphate in the presence of the chelating mixture, resulted in the disappearance of this additional peptide band and a general simplification of the picture towards that shown by the rat liver extracts. NEM, as before, caused movements in the positions of several components.

Electropherograms of extracts of tumours have shown pictures similar to those of rat liver. Again NEM has caused alterations in the positions of several bands (Fig. 5). The band due to the thiol peptide of Neish and Rylett (1961) has also been found in acid extracts of tumours.

From the above it appears that in all cases there are a number of components which react with NEM and, therefore, are to be considered as –SH containing compounds.

### DISCUSSION

The evidence from the above experimental work confirms the view that glutathione is not the only –SH containing component of acid extracts of tissues. Further, since methods of estimation of –SH are not specific for any one compound (even the glyoxalase method is subject to this proviso (Patterson and Lazarow, 1954)) measurements of –SH in acid extracts are not measurements of glutathione. It would be more accurate to refer to acid soluble –SH and, to specify the acid used for extraction processes.

From the densitometer traces of electropherograms it is possible to obtain the relative percentages of the various components by measuring the areas under the peaks and relating them to the total area measured. In all the cases illustrated above it is evident that the area under the GSH peak never represents more than one third of the total area under those peaks which are subject to alteration by reaction with NEM. As reaction with NEM must be regarded as a criterion of the presence of available –SH groups it follows that if all these groups are measured in the normal –SH measurement then the true glutathione component probaby never exceeds one third of the measured thiol content. This also means that measurements of fluctuations in glutathione (so called) content could in fact be measurements of alterations in some other –SH bearing component.

This work has fully confirmed the presence of an additional component in guinea-pig liver and animal tumours as found by Neish and Rylett (1960, 1961). No further evidence as to the nature of this peptide is available but in its properties it does appear to strongly resemble the glutathione derivative $G.SSO_3H$ isolated by Waley (1959) from calf lens.

The nature of the other components which have been found on electropherograms to contain –SH groups is also unknown. Ergothioneine is not represented amongst them since this compound does not stain with ninhydrin. Homocysteine might be expected to occur in tissues as the result of demethylation of methionine but its presence has never been proved. The prominent –SH containing band moving at about half the rate of the glutathione occurs on the electropherograms in a position consistent with it being homocysteine, but this cannot be taken as evidence of identity. No indications exist as to the identity of the other components present.

### SUMMARY

1. Measurements of the –SH content of metaphosphoric, sulphosalicylic and trichloroacetic acid extracts of various tissues and animal tumours have been compared and found to differ.

2. Partial or complete saturation of such acid extracts with ammonium or sodium sulphate has been found to alter the apparent –SH content.

3. Electrophoretic studies of such acid extracts indicate the presence of several –SH containing components.

4. The true glutathione –SH in an acid extract of tissue is estimated as being not more than one third of the total free –SH present.

The expenses of this work were defrayed from a block grant from the British Empire Cancer Campaign.

### REFERENCES

CALCUTT, G.—(1961) *Brit. J. Cancer*, **15**, 673.
*Idem* AND COATES, JOAN.—(1961) *Ibid.*, **15**, 360.
*Idem* AND DOXEY, D.—(1959) *Exp. Cell. Res.*, **17**, 542.
*Iidem* AND COATES, JOAN.—(1960) *Brit. J. Cancer*, **14**, 746.—(1961) *Ibid.*, **15**, 149.
CHINARD, F. P. AND HELLERMAN, L.—(1954) In ' Methods of Biochemical Analysis '. Vol. 1. Edited by Glich, D. New York (Interscience Publishers, Inc.).
HOPKINS, F. G.—(1921) *Biochem. J.*, **15**, 286.
NEISH, W. J. P. AND RYLETT, ANN.—(1960) *Brit. J. Cancer*, **14**, 737.—(1961) *Ibid.*, **15**, 630.
PATTERSON, J. W. AND LAZAROW, A.—(1954) in ' Glutathione, a Symposium '. New York (Academic Press).
SAKAI, H. AND DAN, K.—(1959) *Exp. Cell Res.*, **16**, 24.
WALEY, S. G.—(1959) *Biochem. J.*, **71**, 132.